# Acute kidney injury and chronic kidney disease in umbilical cord blood transplant recipients

**DOI:** 10.3389/fonc.2023.1186503

**Published:** 2023-05-16

**Authors:** Paolo Lopedote, Elisabetta Xue, Julie Chotivatanapong, Emily C. Pao, Chiara Wychera, Ann E. Dahlberg, Laurel Thur, Laura Roberts, Kelsey Baker, Ted A. Gooley, Sangeeta Hingorani, Filippo Milano

**Affiliations:** ^1^ Department of Medicine, St. Elizabeth’s Medical Center, Boston University, Boston, MA, United States; ^2^ Hematology and Bone Marrow Transplant Unit, IRCCS San Raffaele Scientific Institute, Milano, Italy; ^3^ Clinical Research Division, Fred Hutchinson Cancer Center, Seattle, WA, United States; ^4^ Department of Pediatrics, University of Washington, Seattle, WA, United States

**Keywords:** cord blood transplantation (CBT), allogenic stem cell transplantation, acute kidney injury (AKI), chronic kidney disease (CKD), bilirubin, steroid, graft versus host disease (GVHD)

## Abstract

**Introduction:**

Acute kidney injury (AKI) is a frequent early complication post hematopoietic stem cell transplant (HSCT), associated with high morbidity and mortality. Cord blood transplant (CBT) recipients are potentially exposed to more nephrotoxic insults, compared to patients undergoing HSCT from other donor sources. We aimed to identify risk factors for AKI in patients undergoing CBT. We also aimed to identify the impact of AKI on chronic kidney disease (CKD) and survival outcomes by one-year post-CBT.

**Methods:**

Adults and children who underwent a first CBT at our Institution were retrospectively evaluated. AKI was staged according to Kidney Disease Improving Global Outcomes (KDIGO) definitions. Cox regression models were used to estimate the association of demographic factors and post-CBT parameters with the cause-specific hazard of AKI.

**Results:**

We identified 276 patients. Median age was 32 years, 28% (77/276) were children (<18 years) and 129 (47%) were white. A myeloablative conditioning regimen was administered to 243 patients (88%) and 248 (90%) received cyclosporine for GVHD prophylaxis. One-hundred and eighty-six patients (67%) developed AKI by day 60 post-transplant, with 72 (26%) developing severe AKI (stage 2 and 3). In a multivariable analysis, each increase in bilirubin level of 1 mg/dL was associated with a 23% increase in the risk of severe AKI (adjusted HR 1.23, 95% CI 1.13 – 1.34, p<.0001). Conversely, systemic steroid administration appeared to be protective of severe AKI (unadjusted HR 0.36, 95% CI 0.18 – 0.72, p=.004) in a univariate model . Two-hundred-forty-seven patients were evaluable at the one-year time point. Among those, 100 patients (40%) developed CKD one-year post-CBT. Severe AKI was associated with a higher hazard of non-relapse mortality (adjusted HR=3.26, 95% CI 1.65-6.45, p=.001) and overall mortality (adjusted HR=2.28, 95% CI 1.22-4.27, p=.01).

**Discussion:**

AKI is a frequent complication after CBT and is associated with worse outcomes. Questions remain as to the mechanism of the protective role of steroids on kidney function in the setting of CBT.

## Introduction

Cord blood transplantation (CBT) is an established alternative to traditional hematopoietic stem cell transplantation (HSCT) in patients who lack a suitable Human Leukocyte Antigen (HLA)-matched donor. Traditional advantages of CBT include higher degree of HLA mismatches allowance, ease of collection and low rate of chronic graft versus host disease (cGVHD) (1, 2). However, CBT recipients are at higher risk of non-relapse mortality (NRM) when compared to those undergoing HSCT from related or unrelated HLA-matched donors (3, 4). This increased NRM mostly derives from a delayed engraftment and the lack of immunological memory of CB T-cells, resulting in a higher incidence of opportunistic infection (2, 5).

Despite significant improvements in HSCT, acute kidney injury (AKI) and chronic kidney disease (CKD) remain common complications post-HSCT (6, 7) and are associated with an increased mortality risk (8–14). Rates of developing AKI range from 10% in autologous HSCT recipients up to 73% in allogeneic HSCT recipients (6, 7). Causes of AKI in this population vary, and can be graft-related, infection-related, GVHD-related, or iatrogenic. Transplant-associated thrombotic microangiopathy (TA-TMA) is also an increasingly recognized cause of AKI following allogenic HSCT, whose hallmarks are endothelial cell injury and complement cascade activation (15). While overall survival is comparable to that observed after HSCTs from matched-unrelated donor and possibly matched-sibling donor (16), CBT recipients are exposed to more nephrotoxic insults, including infectious complications (e.g., gram-negative rods, cytomegalovirus, BK virus), and consequently antibiotics and anti-viral agents (6, 17–20). A recent study by Gutgarts et al. found a very high rate of AKI (83%) in the first 100 days post-transplant in 153 adults who received CBT +/- haploidentical cell infusion after reduced intensity conditioning regimen (21). Researchers in this study identified African ancestry, concomitant haploidentical CD34+ cell infusion, nephrotoxic medication, ICU admission, and lower albumin level at baseline as risk factors for developing AKI (21).

Hence, we decided to retrospectively evaluate the rate of AKI in a large cohort of patients receiving CBT at the Fred Hutchinson Cancer Center (FHCC) and compare to the available studies. Furthermore, we decided to explore risk factors associated with the development of AKI, as well as the impact of AKI on CKD development. Unlike previous work, our cohort was represented by both adult and pediatric CBT recipients, receiving both myeloablative (MAC) and non-myeloablative conditioning (NAC).

## Subjects and methods

### Study design and patient selection

Adult and pediatric patients undergoing first single unit or double units CBT at the FHCC between August 2006 and June 2018 on research protocols approved by the Center’s Institutional Review Board were eligible for this retrospective analysis. Patients diagnosed with multiple myeloma and non-malignant diseases were excluded. CBT was considered when HLA-compatible related or unrelated donors were unavailable. Selected CB units were required to be matched to the recipient at ≥ 4 of the 6 HLA loci based on intermediate resolution typing at HLA-A and –B and allele-level for HLA-DRB1 typing; for double units CBT recipients, units must be a minimum match of 3 of the 6 HLA loci. No patients received concomitant haploidentical cell infusion. Hematopoietic cell transplantation-specific comorbidity index was calculated for all patients ≥ 45 years-old (22).

Patients received either NAC or MAC regimen (**Table 1**). NAC regimens included fludarabine (Flu) 150 mg/m2, cyclophosphamide (Cy) 50 mg/kg, and total body irradiation (TBI) 200 – 300 cGy. MAC regimens included one of the following regimens: Flu, Cy and high dose TBI (1230-1300 cGy) or Treosulfan (14 or 10 g/m2), Flu and TBI 200 – 300 cGy. In all patients included in the analysis, prophylaxis for GVHD consisted of cyclosporine beginning on day -3 (therapeutic range, 275-350 ng/mL) and continuing for a minimum of 180 days, and mycophenolate mofetil (MMF) beginning on day 0 (starting dose 15 mg/kg i.v. every 8 h; maximum 1500 mg every 8 h) and administered until at least day 30-45 or potentially longer in the presence of active GVHD. Pre-engraftment syndrome (PES) data were not collected. Acute GVHD (aGVHD) was graded using Mount Sinai Acute GVHD International Consortium criteria based on stages of organ involvement and categorized as grades 0–4 (23). Time to neutrophil engraftment was defined as the first of two consecutive days with an absolute neutrophil count (ANC) of 0.5 x 10^9^ per liter or higher, and time to platelet engraftment as the first of 7 consecutive days with an unsupported platelet count of 20 x 10^9^ per liter or higher. Post-transplant granulocyte-colony stimulating factor (G-CSF) was given until the absolute neutrophil (ANC) recovery to > 2.5 x 10^9^/L was stable for three days, and then reduced to as needed while maintaining an ANC > 1.0 x 10^9^/L. Sinusoidal obstruction syndrome was defined using the modified Seattle criteria (24). TA-TMA was defined using the criteria proposed by Cho et al. in 2010 (25).

**Table 1:** Clinical characteristics of the study population.

Variables	Overall no. (%)
Number	276
Age at CBT	
Median years (range)	31.7 (0.6-73.1)
< 18 years	77 (28%)
≥ 18 years	199 (72%)
Sex	
Female	130 (47%)
Male	146 (53%)
Ethnicity	
Caucasian	129 (47%)
Non-Caucasian	138 (50%)
Unknown	9 (3%)
Patient CMV serostatus	
Positive	180 (65%)
Negative	91 (33%)
Missing data	5 (2%)
Underlying Disease	
Acute Leukemia	227 (82%)
Others^†^	49 (18%)
Disease severity ^¶^	
Intermediate	167 (61%)
High	109 (39%)
HCT-CI	
0	25 (9%)
1-2	36 (13%)
>3	50 (18%)
Not Assessed per Age	149 (54%)
Not Available	16 (6%)
Type of CBT	
Single CBT	58 (21%)
Double CBT	218 (79%)
Conditioning regimen	
NAC ^§^	33 (12%)
MAC	243 (88%)
Use of TBI	
Low dose	104 (38%)
High dose ^‡^	172 (62%)
GVHD prophylaxis^₤^	
Cyclosporine-based	248 (90%)
Tacrolimus-based	28 (10%)

CBT, cord blood transplant; CMV, cytomegalovirus; HCT-CI, hematopoietic cell transplantation-specific comorbidity index; NAC, non-myeloablative conditioning; MAC, myeloablative conditioning; TBI, total body irradiation; GVHD, graft versus host disease.

^†^Other diseases included myelodysplastic syndromes (n=31), chronic myeloid leukemia (n=7), chronic lymphocytic leukemia (n=2), non-Hodgkin lymphoma (n=4), non-specified lymphoproliferative disease (n=3), polycythemia vera (n=1), unknown (n=1).

^¶^High disease severity was defined as active relapses, complete remission ≥ 2, refractory anemia with blasts excess or multilineage dysplasia in active disease; all other cases were considered with intermediate disease severity.

^§^NAC regimens were all based on Fludarabine, Cyclophosphamide and low dose Total Body Irradiation (TBI) Myeloablative conditioning regimens included Fludarabine-Cyclophosphamide plus high-dose TBI or Treosulfan-Fludarabine plus low dose TBI.

^‡^Patients who received high dose TBI were given a total dose of 1200 cGy or 1320 cGy.

^₤^Only 6 patients (2%) received also sirolimus, thus its effect was excluded from the analysis.

### Data collection

We retrospectively reviewed medical records and the clinical database of CBT recipients. Standard biochemistry panel, including kidney and liver function, is collected daily until hospital discharge and then at each follow-up appointment.

We estimated the glomerular filtration rate (eGFR) using the serum creatinine based Schwartz formula for children (age ≤18) and the CKD Epidemiology Collaboration (CKD-EPI) creatinine-only equation for adults (26, 27). Cytomegalovirus (CMV) reactivation was defined as the presence of any viral load and CMV viral loads were monitored routinely per standard practice, whereas testing for serum adenovirus (AdV) and serum BK virus was performed as clinically indicated at the discretion of the attending physician. We collected data regarding the use of potential nephrotoxic antimicrobial drugs, namely vancomycin, amphotericin, and gentamicin within 60 days from transplantation. Any positive CMV level triggered the initiation of treatment with either foscarnet or ganciclovir. Calcineurin inhibitor (CNI) level monitoring was performed according to institutional guidelines.

### Definition

We defined AKI according to the Kidney Disease Improving Global Outcomes (KDIGO) guidelines from 2012; AKI is defined as a 0.3 mg/dL increase in baseline serum creatinine within 48 hours or an increase of 1.5 times baseline within 7 days. Stage 1 AKI: an increase in serum creatinine of 1.5-1.9 times baseline; stage 2: an increase of 2.0-2.9 times baseline; and stage 3: an increase in serum creatinine of ≥ 3.0 times baseline or a serum creatinine absolute value of ≥ 4.0 mg/dL (28). We defined severe AKI as stage ≥2 within the first 60 days post-transplant (29).

CKD was defined as a >20% decrease in eGFR from baseline to one-year post-CBT (day 365 +/- 90 days) (30). Bilirubin level was monitored in the first 28 days from transplant.

### Statistical analysis

Baseline demographics of interest included age at transplant, sex, ethnicity, underlying diagnosis, donor sex, donor CMV status, number of CB units, and conditioning regimen.

Primary endpoints of interest were AKI of any stage by 60 days post CBT, severe AKI, overall mortality (OM) and non-relapse mortality (NRM).

Cox regression models were used to estimate the association of demographic factors and post-CBT parameters with the cause-specific hazard of AKI. Univariate models were used to estimate the impact of factors on risk of developing AKI, and a multivariable model was constructed from factors with a p-value such that p<.05. At least 10 events per degree of freedom were required in the multivariable model. Variables [e.g., occurrence of aGVHD, receipt of medications, laboratory values (such as bilirubin level)] that occurred or were assessed following CBT were treated as time-dependent covariates. Continuous variables were modeled as continuous linear covariates (in some cases after a log-transformation). The impact of AKI on OM and NRM were assessed using Cox regression with AKI modeled as a time-dependent covariate, and models were adjusted for age, sex, CMV serostatus, disease severity (intermediate vs high).

CKD incidence at one-year post-CBT was examined as a secondary endpoint. Patients who did not have an available creatinine value within 90 days of day 365 were excluded from the analysis. We compared proportions of patients who developed CKD among those who previously had AKI to the proportion who developed CKD among those who did not have AKI using a chi-square test.

All listed p-values are two-sided, and no adjustments were made for multiple comparisons. Statistical analyses were performed using SAS Statistical Software Version 9.4.

## Results

### Patient characteristics

A total of 276 adults and pediatric patients underwent a first CBT between August 2006 and June 2018 at FHCC. Baseline patient demographics and transplant characteristics are shown in **Table 1**. The median age at transplant was 32 (range, 0-73). Acute leukemia was the most frequent diagnosis (227 patients, 82%). Of the 127 patients eligible for screening, HCT-CI was available for 111 patients (87%, median 2, range 0-8). Two-hundred and forty-three patients (88%) received a MAC regimen and 218 (79%) received a double unit CBT.

The median time to ANC engraftment was 19 days (range 6 – 89) and the median time of platelet engraftment was 34 days (range 9 – 99). Seventy-eight percent (215/276) of patients developed aGVHD, 177 of whom (64%) had aGVHD prior to day 60. Twenty percent (52/276) experienced grade 3-4 aGVHD. In 260 (94%) patients we were able to evaluate the occurrence of SOS and TA-TMA. Only 3 and 4 patients were diagnosed with SOS and TA-TMA, respectively, and because of their rarity they were not included in the analysis. Patients were followed for a median of 25.4 months (range 0.2 – 135.4 months); death from any cause occurred in 102 patients (37%). Vancomycin, gentamycin, and amphotericin were administered in 145 (53%), 33 (12%) and 14 (5%) patients, respectively. A positive PCR on blood for CMV and BK virus was documented in 126 (46%) and 41 (15%) of patients, respectively. AdV detection in blood occurred in 5 (2%) patients and because of its rarity was not included in the analysis.

### Incidence of AKI post-CBT

Among the 276 patients 67% (186/276) had AKI of any stage. Stage 1 AKI occurred in 41% (114/276), stage 2 in 16% (43/276), stage 3 in 11% (29/276) of patients; 72 of the 276 (26%) patients had severe AKI (defined as ≥ stage 2). The median time to AKI, defined as the time that the probability of AKI reached or crossed 50%, was 39 days. The timing of AKI among those who developed AKI ranged from 0 to 60 days, with the median time of AKI occurrence being 22 days.

### Risk factors for AKI in CBT recipients

The associations of demographic data at baseline and post-CBT parameters with the risk of AKI are summarized in **Table 2** (AKI of any stage) and **Table 3** (severe AKI), including both univariate and multivariable models.

**Table 2:** Univariate and Multivariable Cox Regression Models for risk factors for Day 60 AKI of any stage.

Covariate	Categories	HR (95% CI)	*P*	aHR (95% CI)	*P*
Age (years) – per one-year increase					
		1.02 (1.01-1.03)	<.0001	1.00 (0.99-1.01)	.73
Sex					
	Female	1		1	
	Male	0.73 (0.55-0.97)	.03	0.67 (0.47-0.96)	.03
Conditioning regimen					
	MAC	1			
	NAC	1.27 (0.83-1.95)	.27		
Baseline creatinine – per 1 Unit increase					
		2.93 (1.78-4.83)	<.0001	2.04 (0.93-4.47)	.08
Acute GVHD^‡^					
	Grade 0-1	1		1	
	Grade 2-4	0.38 (0.26-0.55)	<.0001	0.43 (0.28-0.66)	.0001
Acute Skin GVHD^‡^					
	Grade 0-1	1			
	Grade 2-4	0.5 (0.25-0.72)	.0002		
Acute Liver GVHD^‡^					
	Grade 0-1	1			
	Grade 2-4	1.0 (0.47-2.15)	>.99		
Acute Gut GVHD^‡^					
	Grade 0-1	1			
	Grade 2-4	0.27 (0.12-0.62)	.002		
Systemic steroid administration^‡^					
		0.53 (0.36-0.78)	.001		
Vancomycin administration^‡^					
		2.09 (1.4-3.13)	.0003	1.63 (1.03-2.58)	.04
Amphotericin administration^‡^					
		5.24 (1.66-16.51)	.005	1.41 (0.33-6.05)	.65
Gentamycin administration^‡^					
		0.87 (0.21-3.57)	.85		
Cyclosporine^¶^ – per 100 Unit increase					
		1.24 (1.14-1.35)	<.0001	1.23 (1.13-1.34)	<.0001
Tacrolimus^¶^ – per 1 Unit increase					
		1.11 (0.7 – 1.74)	.66		
Bilirubin^¶^ – per 1 Unit increase					
		1.24 (1.15 - 1.34)	<.0001	1.13 (1.02-1.26)	.03
Log(CMV) ^¶^ – per 1 Unit increase					
		1.02 (0.85-1.23)	.81		
Log(BK) ^¶^ – per 1 Unit increase					
		0.73 (0.53-1.02)	.07		

AKI, acute kidney injury; HR, Hazard Ratio; aHR, adjusted HR; NAC, non-myeloablative conditioning; MAC, myeloablative conditioning; GVHD, graft versus host disease; CMV, cytomegalovirus.

^‡^These variables were modeled as time-dependent indicator variables.

^¶^These variables were modeled as time-dependent continuous linear variables.

**Table 3:** Univariate and Multivariable Cox Regression Models for risk factors for Day 60 severe AKI.

Covariate	Categories	HR (95% CI)	*P*	aHR (95% CI)	*P*
Age (years) – per one-year increase					
		1.0 (0.99-1.02)	.53	1.00 (0.98-1.02)	.95
Sex					
	Female	1		1	
	Male	0.58 (0.36-0.93)	.02	0.66 (0.37-1.16)	.15
Conditioning regimen					
	MAC	1			
	NAC	0.53 (0.21-1.32)	.17		
Baseline creatinine – per 1 Unit increase					
		0.9 (0.37-2.18)	.82		
Acute GVHD^‡^					
	Grade 0-1	1		1	
	Grade 2-4	0.54 (0.3-0.96)	.04	0.53 (0.26-1.10)	.09
Acute Skin GVHD^‡^					
	Grade 0-1	1			
	Grade 2-4	0.65 (0.36-1.18)	.16		
Acute Liver GVHD^‡^					
	Grade 0-1	1			
	Grade 2-4	2.73 (1.17-6.4)	.02		
Acute Gut GVHD^‡^					
	Grade 0-1	1			
	Grade 2-4	0.65 (0.23-1.8)	0.4		
Systemic steroid administration^‡^					
		0.36 (0.18-0.72)	.004		
Vancomycin administration^‡^					
		0.23 (0.06-0.95)	.04		
Gentamycin administration^‡^					
		2.1 (0.5-8.85)	.31		
Cyclosporine^¶^ – per 100 Unit increase					
		1.19 (1.03-1.37)	.02	1.18 (1.02-1.37)	.02
Tacrolimus^¶^ – per 1 Unit increase					
		1.21 (0.9-1.62)	.2		
Bilirubin^¶^ – per 1 Unit increase					
		1.3 (1.18-1.43)	<.0001	1.23 (1.13-1.34)	<.0001
Log(CMV) ^¶^ – per 1 Unit increase					
		1.37 (1.07-1.76)	.01	1.35 (0.98-1.85)	.06
Log(BK) ^¶^ – per 1 Unit increase					
		0.58 (0.32-1.05)	.07		

AKI, acute kidney injury; HR, Hazard Ratio; aHR, adjusted HR; NAC, non-myeloablative conditioning; MAC, myeloablative conditioning; GVHD, graft versus host disease; CMV, cytomegalovirus.

Amphotericin administration was not considered in this model due to the limited number of patients.

^‡^These variables were modeled as time-dependent indicator variables.

^¶^These variables were modeled as time- dependent continuous linear variables.

Among several factors of note in **Table 2**, the multivariable model showed that each 1mg/dL increase in the post-CBT bilirubin level led to an increased risk of AKI of any stage (HR 1.13, 95% CI 1.02 – 1.26), as did the use of vancomycin (adjusted HR (aHR) 1.63, 95% CI 1.03 – 2.58) and each 100 ng/mL increase in the level of cyclosporine (aHR 1.23, 95% CI 1.13 – 1.34). Presence of grades II-IV acute GVHD and male sex showed reduced risk of AKI of any stage, compared to grades 0-I and female sex, respectively (aHR 0.43, 95% CI 0.28 – 0.66: aHR 0.67, 95% CI 0.47 – 0.96).

For severe AKI, increasing bilirubin following CBT led to a higher risk of severe AKI in the multivariable model (aHR 1.23, 95% CI 1.13 – 1.34), while systemic steroid use led to a lower risk of severe AKI (HR 0.36, 95% CI 0.18 – 0.72) in a univariate model.

### Overall survival and non-relapse mortality

Day-100 and day-365 estimates of overall survival were 90% (95% CI: 86% - 93%) and 71% (95% CI: 65% - 76%), respectively. We evaluated the impact of AKI on the risk of OM and NRM after adjusting for age, sex, CMV serostatus, and disease severity (**Table 4**). 

**Table 4:** Multivariable cox regression models for overall mortality and non-relapse mortality.

Covariate	Categories	aHR (95% CI) for OM	*P*	aHR (95% CI) for NRM	*P*
Age (years) – per one-year increase					
		1.03 (1.02-1.04)	<.0001	1.03 (1.01-1.03)	0.0001
Sex					
	Female	1		1	
	Male	1.35 (0.9-2.01)	.14	1.43 (0.86-2.38)	.17
CMV serostatus					
	Negative	1		1	
	Positive	1.02 (0.66-1.56)	.93	1.25 (0.7-2.22)	.45
Disease severity					
	Intermediate	1		1	
	High	1.32 (0.89-1.96)	.17	1.09 (0.66-1.83)	.73
Acute Kidney Injury					
	0-1	1		1	
	2-3	2.28 (1.22-4.27)	0.01	3.26 (1.65-6.45)	.001

aHR, adjusted HR; OM, overall mortality; NRM, non-relapse mortality; CMV, cytomegalovirus.

**Figure 1:**
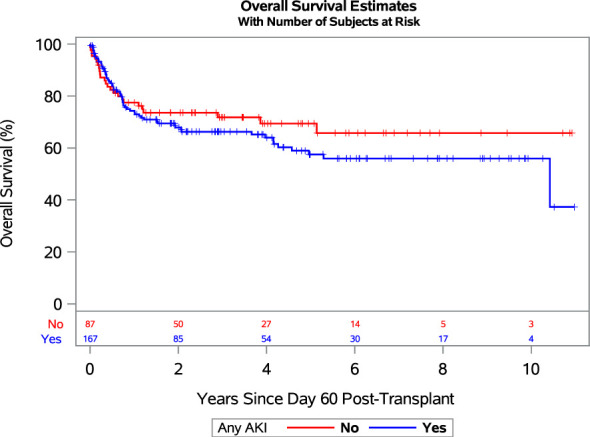
Landmark analysis showing overall survival since day 60 post-transplant for patients who developed any grade AKI (blue line) and no AKI (red line). Abbreviations: AKI, acute kidney injury.

The adjusted HRs for OM and NRM among patients with severe AKI relative to those without AKI or stage-1 AKI were 2.28 (95% CI: 1.22-4.27) and 3.26 (95% CI: 1.65-6.45), respectively. While these regression models treat AKI as a time-dependent covariate, for graphical purposes we performed a landmark analysis at day 60. **Figure 1** shows OS after day 60 among those who survived to day 60 (254 of 276 patients), grouping patients into those who developed AKI of any stage in the first 60 days post-CBT and those who did not.

### Incidence of CKD and association with AKI

Of the 276 patients who underwent CBT, 70 (25%) died within 1 year from transplant, while 29 (11%) patients did not have available data to calculate eGFR at one year (or within the 90-day window surrounding one year). Among 247 patients whose CKD status at one year was known (including those who died before one year), 100 (40%) had CKD. Among 164 patients with AKI of any stage, 66 (40%) developed CKD at one year, and among 83 patients who did not have AKI, 34 (41%) developed CKD. These results were qualitatively similar when the proportion of CKD was compared between those with severe AKI to those with no AKI or stage-1 AKI. Namely, among 69 patients with severe AKI, 26 (38%) developed CKD at one year compared to 74 of 178 (42%) patients who had no AKI or stage-1 AKI.

If one instead restricts the above analysis to the 178 patients who survived to the one-year time point and whose CKD status at one year was known, 116 patients had AKI of any stage. Among these 116 patients with AKI, 66 (57%) went on to develop CKD at one year compared to 34 of 62 (55%) patients without AKI. Similarly, 26 of 44 (59%) patients with severe AKI had CKD at one year compared to 74 of 134 (55%) patients without AKI or with stage-1 AKI.

## Discussion

To our knowledge, this is the largest study evaluating factors associated with AKI and its impact on patient outcomes after CBT in both adult and pediatric recipients. We found that both any AKI (67%) and severe AKI (26%) occur frequently in our population. We also found that severe AKI increases the risk of NRM and OM relative to patients who did not develop AKI or had only stage-1 AKI. Increasing bilirubin level following CBT led to an increased risk of both any grade and grade ≥2 AKI. In contrast, steroid use reduced the risk of severe AKI in a univariate model. The proportion of patients with AKI who developed CKD at one year was similar regardless of AKI status, and this held true even after excluding from analysis patients who died before one year.

The relatively high rate of AKI observed in our cohort is consistent with the literature on HSCT. In a previous study from our group, the incidence of AKI ranged between 50% in NAC and 73% in MAC allogenic transplants (6). Similarly, in a more recent retrospective analysis of HSCT recipients (other than CBT) ranging from 19 to 79 years old (14), AKI of any grade and severe AKI occurred in 64% and 30% of patients, respectively. However, when focusing solely on the CBT recipients, a higher frequency of any stage AKI (83%) and, of severe AKI (54%) was detected by Gutgarts et al. (21). The reasons for this discrepancy could be several. In that study, vancomycin was administered prophylactically to all patients and further nephrotoxic medications (other than GVHD prophylaxis), were given to an additional 24% of patients (21). Conversely, in our cohort, vancomycin was administered to 53% of patients and its use was associated with a higher risk of AKI of any stage. Additionally, only a minority of patients received gentamicin and amphotericin (12% and 5%, respectively), which might partially explain the apparent lack of association between exposure to those two agents and kidney injury. Furthermore, none of our patients received concomitant haploidentical CD34+ cell infusion, which was identified as a risk factor in a multivariable analysis by Gutgarts et al (21).

In our analysis, BK reactivation did not seem to increase the risk of AKI, although such an association has been described (31); this might be partially explained by the small numbers of infections by day 60 (15%), and by the limits of data capture, since BK viremia was not routinely monitored. AdV reactivation occurred in 2% of patients and was thus excluded from the statistical analysis. As expected, we observed a high rate of CMV reactivation, which was associated with a numerical increase in the risk of severe AKI, although it did not reach statistical significance at the multivariable analysis. The direction of this observation is consistent with previous studies (30, 32) where CMV viremia was associated with AKI or with a decline of GFR over time. The lack of statistical significance of our finding might be explained by the difference in the study populations and by fact that treating CMV reactivation at any level of viral load has resulted in a lower incidence of multiple reactivations and disease (33–35). The recent introduction of letermovir prophylaxis might further decrease the impact of early CMV reactivation on post-HSCT outcomes (33).

The kidney toxicity of cyclosporine has been very well described; however, the results in this HSCT patient population are mixed. The effects of cyclosporine use on the kidney include vasoconstriction of the afferent glomerular arteriole, activation of the renin-angiotensin system, tubulo-interstitial scarring, leading ultimately to a decrease in eGFR (36). Although early studies have pointed at CNIs as a risk factor for AKI after HSCT, more recent studies have failed to find a strong association (11, 12, 30). We observed a demonstrable increase in the risk of AKI of any stage with increased levels of cyclosporine. The risk of severe AKI was also increased with higher levels of cyclosporine, but after adjusting for other factors, the association, while still positive, was closer to the null of no association. Gutgarts and colleagues observed an association in the same direction and suggested a reduction of cyclosporine target level in CBT recipients (21).

In our analysis the intensity of the conditioning regimen did not show a demonstrable association with AKI, and the direction of association was different for AKI of any stage compared to severe AKI. Although historically MAC was reported to be associated with a higher incidence of AKI (6, 37, 38), these studies included stem cell sources other than CB. In Gurgarts’ study, no patients received NAC conditioning and therefore no conclusion could be derived for this subset of patients (21). Notably, a more recent study from the same group (14) identified a higher incidence of AKI among patients receiving NAC conditioning regimens with donor sources other than CB. However, more studies are needed to clarify the association between the type of conditioning regimen and the development of AKI in patients undergoing CBT.

Another risk factor for AKI identified in the current study was increased bilirubin level. Several insults can lead to bilirubin elevation, including iatrogenic toxicity, sepsis with multi-organ failure, and SOS. Previous studies evaluated the association between AKI and elevated bilirubin, either as independent variable or in the context of SOS (39–42). In most of these studies (41, 42), although the direction of the association between bilirubin elevation and AKI was consistent with the one we detected, only SOS was significantly associated at the multivariable analysis. The pathophysiology underlying AKI in patients with SOS appears to be a variant of the hepatorenal syndrome (12, 43, 44). In our study, the diagnosis of SOS was extremely rare. This finding is likely related to not using busulfan in the conditioning regimen for CBT. This study confirms the association between bilirubin elevation and AKI in the CBT setting, where it was not previously described.

The seemingly protective effect aGVHD had on AKI in regression models was unexpected. Given its magnitude, we hypothesized that not aGVHD itself, but rather its treatment with systemic steroids could be playing a role in protecting kidney function. That said, since presence of aGVHD and receipt of steroids are obviously strongly correlated, we only allowed presence of aGVHD in the multivariable regression models. Although no consensus has been reached regarding the kidney involvement in the GVHD process, kidney lymphocyte infiltration and local upregulation of inflammatory genes have been described in animal models of aGVHD (45–47). Additionally, documentation of glomerulitis, renal tubulitis, and peritubular capillaritis have been observed in HSCT-recipients with cGVHD, suggesting a possible direct inflammation of kidney tissues (48). Previous reports have also described responsiveness to steroid and/or other immunosuppressants used to treat nephrotic syndrome (including minimal change disease and membranous glomerulonephritis) occurring as late post-HSCT complications, likely as part of cGVHD syndrome (49–51). However, reports on kidney aGVHD in humans are more limited, mainly due to challenges in obtaining kidney biopsies and lack of established histopathologic criteria. In a recent prospective case series on 8 HSCT patients, a kidney biopsy was performed to aid in the management of persistent kidney dysfunction, revealing a high frequency of thrombotic microangiopathy and GVHD as a cause of AKI/CKD in this setting (52). Therefore, we suggest that among different causes of AKI, providers should keep in mind a possible GVHD etiology and discuss biopsy when deemed to affect patient management.

The similar frequency of CKD development at one year between those with and without AKI by day 60 was also unexpected. Previous studies identified AKI as a risk factor for subsequent development of CKD, with an incidence of CKD ranging from 16% to 34% among long-term HSCT survivors (10, 14, 21, 53–55). In their study, Gutgarts et al. reported an incidence of 34% of CKD at two years post HSCT and this was more frequent in patients with a previous AKI with eGFR <60 ml/min per 1.73 m (2) and lasting ≥28 days. In our study, the incidence of CKD appeared higher (40%) compared to previous analyses and with similar rates among patients with or without prior AKI. The reasons for such discrepancy and for the lack of association between AKI and CKD are not entirely clear, but could be related to the different definitions of CKD used in previous studies resulting in either over or underestimation of CKD.

There are several limitations to this study, which can be primarily attributed to its retrospective nature. As the FHCC serves a large geographical area, not all CBT recipients return to our center for the one-year visit, leading to a lack of information at this timepoint and thus an incomplete secondary analysis of the association of AKI and CKD by one-year post-CBT. In addition, due to challenges in retrospective data capture, some variables were not collected, such as volume status, duration of nephrotoxic medications, vancomycin levels, and urine studies (e.g., proteinuria). One of the limitations of our study was also the inability of reporting the incidence and timing of PES. The latter was related to the clinical difficulty of discriminating between PES and hyperacute GVHD based on time of onset and type of treatment of these two overlapping entities. Lastly, we used serum creatinine to determine eGFR in the diagnosis of CKD, which may result in an inaccurate estimation in the setting of decreased muscle mass, particularly in children (56).

Overall, we describe a high rate of AKI in a large CBT cohort of pediatric and adult patients. Elevated bilirubin levels significantly increase the risk of AKI, indicating the need to clarify the significance and pathophysiology of this association through further studies. Lastly, we reported for the first time a negative correlation between aGVHD, and the subsequent use of steroids, and the development of AKI. However, prospective analyses are needed to confirm their possibly protective role, while histopathological data might shed light on the underlying pathophysiological mechanism.

## Data availability statement

The raw data supporting the conclusions of this article will be made available by the authors, without undue reservation.

## Ethics statement

The studies involving human participants were reviewed and approved by Fred Hutchinson Cancer Research Center Institutional Review Board. Written informed consent to participate in this study was provided by the participants’ legal guardian/next of kin.

## Author contributions

FM and SH contributed equally to the manuscript and share senior authorship. FM, SH, TG, and KB conceived the study. KB and TG analyzed the results. PL and EX wrote the initial draft of the manuscript. All authors contributed to the article and approved the submitted version. 
